# Supplementary material for: Isolation and Cultivation of a New Isolate of BTV-25 and Presumptive Evidence for a Potential Persistent Infection in Healthy Goats
Source: Viruses. 2020 Sep 4;12(9):983. doi: 10.3390/v12090983 (PMC7552037; doi:10.3390/v12090983)
Supplement: Supplementary file 1 [file viruses-12-00983-s001.pdf]

**Supplementary material**

**S1. All BTV-25 specific RT-qPCR (Cq value) and cELISA (S/N%) results of the goat herd over time.** Goats were considered positive for BTV-25 specific RT-qPCR when Cq <40 were measured. The cut-off for the cELISA was 50% and serum samples with a ratio S/N% ≤ 50% were scored positive.

| | 02/2015 | | '07/08/2018 | | 04/09/2018 | | 03/12/2018 | | 16/05/2019 | | '08/10/2019 | |
|---|---|---|---|---|---|---|---|---|---|---|---|---|
| **goat** | **PCR** | **ELISA** | **PCR** | **ELISA** | **PCR** | **ELISA** | **PCR** | **ELISA** | **PCR** | **ELISA** | **PCR** | **ELISA** |
| **Negative results in RT-qPCR and in cELISA** | | | | | | | | | | | | |
| 1 | - | - | no Cq | 101 | no Cq | 69 | no Cq | 91 | no Cq | 128 | no Cq | 117 |
| 3 | - | - | no Cq | 70 | no Cq | 70 | no Cq | 50 | no Cq | 82 | no Cq | 109 |
| 5 | - | - | no Cq | 110 | no Cq | 95 | no Cq | 101 | no Cq | 123 | no Cq | 137 |
| 9 | - | - | no Cq | 114 | no Cq | 115 | no Cq | 111 | no Cq | 131 | no Cq | 118 |
| 12 | - | - | no Cq | 119 | no Cq | 109 | no Cq | 124 | no Cq | 126 | no Cq | 143 |
| 20 | - | - | no Cq | 90 | no Cq | 90 | no Cq | 100 | no Cq | 90 | no Cq | 95 |
| 28 | - | - | no Cq | 107 | no Cq | 88 | no Cq | 99 | no Cq | 118 | no Cq | 120 |
| 29 | - | - | no Cq | 117 | no Cq | 97 | no Cq | 103 | no Cq | 120 | - | - |
| 30 | - | - | no Cq | 90 | no Cq | 84 | no Cq | 93 | - | - | - | - |
| 32 | - | - | no Cq | 95 | no Cq | 82 | no Cq | 92 | no Cq | 102 | no Cq | 108 |
| 33 | - | - | no Cq | 124 | no Cq | 123 | no Cq | 121 | no Cq | 126 | no Cq | 132 |
| 36 | - | - | no Cq | 95 | no Cq | 95 | - | - | - | - | - | - |
| 37 | - | - | no Cq | 69 | no Cq | 71 | no Cq | 83 | no Cq | 100 | no Cq | 104 |
| 42 | - | - | no Cq | 73 | no Cq | 69 | no Cq | 74 | no Cq | 101 | no Cq | 93 |
| 47 | - | - | no Cq | 91 | no Cq | 79 | no Cq | 83 | no Cq | 121 | no Cq | 122 |
| 49 | - | - | no Cq | 80 | no Cq | 71 | no Cq | 79 | no Cq | 82 | no Cq | 94 |
| 50 | - | - | no Cq | 61 | no Cq | 66 | no Cq | 66 | - | - | - | - |
| 52 | - | - | no Cq | 89 | no Cq | 93 | no Cq | 87 | - | - | - | - |
| 59 | - | - | no Cq | 128 | no Cq | 111 | no Cq | 107 | no Cq | 116 | no Cq | 123 |
| 63 | - | - | no Cq | 129 | no Cq | 113 | - | - | - | - | - | - |
| 65 | - | - | no Cq | 101 | no Cq | 88 | no Cq | 77 | no Cq | 110 | - | - |
| 74 | - | - | no Cq | 76 | no Cq | 62 | no Cq | 69 | no Cq | 103 | no Cq | 98 |
| 75 | - | - | no Cq | 95 | no Cq | 79 | no Cq | 81 | no Cq | 100 | no Cq | 97 |
| 77 | - | - | no Cq | 114 | no Cq | 89 | no Cq | 79 | - | 125 | no Cq | 113 |
| 82 | - | - | no Cq | 94 | no Cq | 80 | no Cq | 51 | no Cq | 83 | no Cq | 73 |
| 84 | - | - | no Cq | 94 | no Cq | 83 | - | - | - | - | - | - |
| 86 | - | - | no Cq | 99 | no Cq | 87 | no Cq | 86 | no Cq | 116 | no Cq | 116 |
| 87 | - | - | no Cq | 102 | no Cq | 75 | no Cq | 78 | no Cq | 114 | no Cq | 99 |
| 89 | - | - | no Cq | 81 | no Cq | 88 | no Cq | 91 | no Cq | 132 | no Cq | 120 |
| 90 | - | - | no Cq | 70 | no Cq | 85 | no Cq | 79 | no Cq | 119 | no Cq | 94 |
| 92 | - | - | no Cq | 75 | no Cq | 90 | no Cq | 79 | no Cq | 120 | no Cq | 105 |
| 93 | - | - | no Cq | 98 | no Cq | 106 | no Cq | 94 | no Cq | 135 | no Cq | 128 |
| 94 | - | - | no Cq | 93 | no Cq | 100 | no Cq | 109 | no Cq | 145 | no Cq | 138 |
| 98 | - | - | no Cq | 66 | no Cq | 65 | no Cq | 81 | no Cq | 130 | no Cq | 107 |
| 100 | - | - | no Cq | 74 | no Cq | 98 | no Cq | 94 | no Cq | 120 | no Cq | 109 |
| 101 | - | - | no Cq | 71 | no Cq | 85 | no Cq | 96 | no Cq | 105 | no Cq | 101 |
| 103 | - | - | no Cq | 71 | no Cq | 88 | no Cq | 56 | no Cq | 106 | no Cq | 114 |
| 104 | - | - | no Cq | 85 | no Cq | 90 | no Cq | 103 | no Cq | 94 | no Cq | 96 |

| | | | | | | | | | | | | |
|---|---|---|---|---|---|---|---|---|---|---|---|---|
| 105 | - | - | no Cq | 76 | no Cq | 94 | no Cq | 87 | no Cq | 108 | no Cq | 113 |
| 109 | - | - | no Cq | 57 | no Cq | 83 | no Cq | 89 | no Cq | 103 | no Cq | 115 |
| 112 | - | - | no Cq | 66 | no Cq | 102 | no Cq | 91 | no Cq | 107 | no Cq | 107 |
| 113 | - | - | no Cq | 60 | no Cq | 77 | no Cq | 80 | no Cq | 108 | no Cq | 103 |
| 116 | - | - | no Cq | 88 | no Cq | 90 | no Cq | - | no Cq | 125 | no Cq | 126 |
| 117 | - | - | no Cq | 83 | no Cq | 97 | no Cq | 84 | no Cq | 112 | - | - |
| 118 | - | - | no Cq | 57 | no Cq | 67 | no Cq | 85 | - | - | - | - |
| 131 | - | - | - | - | - | - | - | - | no Cq | 98 | no Cq | 100 |
| 133 | - | - | - | - | - | - | - | - | no Cq | 92 | no Cq | 79 |
| 134 | - | - | - | - | - | - | - | - | - | - | no Cq | 68 |
| 135 | - | - | - | - | - | - | - | - | - | - | no Cq | 92 |
| 136 | - | - | - | - | - | - | - | - | - | - | no Cq | 107 |
| **Constantly positive results in RT-qPCR** | | | | | | | | | | | | |
| **Thereof negative in cELISA** | | | | | | | | | | | | |
| 22 | - | - | 38.3 | 96 | 30.0 | 96 | 31.5 | 100 | 34.6 | 122 | 38.7 | 124 |
| 40 | - | - | 36.6 | 75 | 33.8 | 70 | 32.5 | 70 | 31.7 | 89 | - | - |
| 45 | 32.8 | 76 | 37.9 | 84 | 33.5 | 80 | 31.1 | 77 | 35.6 | 95 | 35.0 | 57 |
| 60 | - | - | 36.5 | 109 | 31.4 | 89 | 31.8 | 88 | 30.1 | 101 | 34.6 | 114 |
| 61 | - | - | 36.2 | 70 | 35.8 | 57 | 34.3 | 57 | 34.6 | 75 | 38.4 | 78 |
| 110 | - | - | 35.0 | 68 | 34.1 | 63 | 28.5 | 80 | 33.0 | 104 | 35.2 | 92 |
| 129 | - | - | - | - | - | - | - | - | 30.8 | 84 | 31.7 | 102 |
| **Thereof positive in cELISA** | | | | | | | | | | | | |
| 26 | - | - | 32.3 | 40 | 31.4 | 36 | 34.5 | 46 | 30.1 | 40 | 34.7 | 39 |
| 27 | - | - | 33.5 | 19 | 31.8 | 20 | 36.1 | 18 | - | - | - | - |
| 39 | - | - | 36.5 | 4 | 32.3 | 5 | 32.2 | 4 | 34.0 | 4 | 37.6 | 3 |
| 41 | - | - | 33.9 | 5 | 33.2 | 7 | 33.9 | 5 | - | - | - | - |
| 58 | - | - | 36.5 | 17 | 35.2 | 22 | 35.0 | 21 | 32.9 | 32 | 34.4 | 27 |
| 91 | - | - | 30.2 | 9 | 30.7 | 12 | 30.0 | 9 | 28.9 | 13 | 36.8 | 12 |
| **Thereof variable in cELISA** | | | | | | | | | | | | |
| 24 | 32.2 | 127 | 31.1 | 24 | 30.5 | 35 | 29.3 | 43 | 28.1 | 63 | 36.5 | 74 |
| 51 | - | - | 33.9 | 55 | 32.4 | 56 | 33.5 | 32 | 29.0 | 72 | 34.5 | 72 |
| 85 | - | - | 37.0 | 114 | 31.7 | 89 | 30.0 | 48 | 34.4 | 77 | 35.9 | 54 |
| 108 | - | - | 30.2 | 73 | 30.0 | 75 | 28.5 | 55 | 27.2 | 7 | 32.3 | 5 |
| 111 | - | - | 37.6 | 73 | 34.9 | 78 | 34.3 | 64 | 31.2 | 60 | 34.5 | 47 |
| **Constantly negative in RT-qPCR** | | | | | | | | | | | | |
| **Thereof variable in cELISA** | | | | | | | | | | | | |
| 53 | - | - | no Cq | 86 | no Cq | 36 | no Cq | 87 | - | - | - | - |
| 57 | - | - | no Cq | 73 | no Cq | 71 | no Cq | 69 | no Cq | 44 | no Cq | 8 |
| 68 | - | - | no Cq | 76 | no Cq | 63 | no Cq | 44 | no Cq | 63 | no Cq | 65 |
| 83 | - | - | no Cq | 72 | no Cq | 68 | no Cq | 79 | no Cq | 66 | no Cq | 43 |
| **Thereof positive in cELISA** | | | | | | | | | | | | |
| 55 | - | - | no Cq | 8 | no Cq | 9 | no Cq | 7 | no Cq | 8 | no Cq | 5 |
| **Positive results for individual samples in RT-qPCR** | | | | | | | | | | | | |
| **Thereof constantly negative in cELISA** | | | | | | | | | | | | |
| 4 | - | - | no Cq | 124 | no Cq | 114 | no Cq | 89 | no Cq | 110 | 38.6 | 51 |
| 6 | - | - | no Cq | 122 | no Cq | 101 | no Cq | 91 | no Cq | 124 | 35.7 | 73 |

| | | | | | | | | | | | | |
|---|---|---|---|---|---|---|---|---|---|---|---|---|
| 13 | - | - | no Cq | 106 | no Cq | 104 | no Cq | 108 | no Cq | 116 | 35.5 | 82 |
| 14 | no Cq | 59.1 | 33.8 | 71 | 34.0 | 64 | 32.3 | 68 | 33.5 | 87 | - | - |
| 15 | - | - | 35.3 | 69 | 32.4 | 73 | 31.0 | 80 | no Cq | 118 | 36.2 | 134 |
| 16 | no Cq | 52.9 | 33.5 | 60 | 34.5 | 66 | 33.9 | 67 | 34.9 | 81 | - | - |
| 17 | - | - | 36.0 | 92 | 35.0 | 88 | 31.2 | 99 | 35.6 | 97 | no Cq | 104 |
| 18 | - | - | no Cq | 62 | 37.8 | 68 | no Cq | 64 | 35.8 | 86 | - | - |
| 19 | - | - | no Cq | 105 | no Cq | 99 | no Cq | 88 | 35.2 | 125 | no Cq | 100 |
| 23 | - | - | 36.9 | 80 | 37.0 | 80 | no Cq | 88 | no Cq | 111 | no Cq | 68 |
| 35 | - | - | 36.0 | 97 | 34.3 | 104 | no Cq | 111 | 36.5 | 124 | no Cq | 140 |
| 38 | - | - | no Cq | 122 | no Cq | 128 | 31.9 | 56 | no Cq | 55 | no Cq | 73 |
| 44 | - | - | no Cq | 117 | 36.3 | 100 | no Cq | 107 | no Cq | 135 | no Cq | 132 |
| 56 | - | - | no Cq | 116 | no Cq | 99 | 33.6 | 85 | no Cq | 114 | no Cq | 116 |
| 62 | - | - | 35.6 | 104 | 31.8 | 102 | 36.4 | 101 | 29.4 | 106 | no Cq | 112 |
| 64 | - | - | 35.7 | 87 | no Cq | 78 | 34.7 | 74 | - | - | - | - |
| 73 | - | - | no Cq | 91 | 36.1 | 78 | 33.0 | 64 | 31.1 | 77 | - | - |
| 78 | - | - | 35.2 | 132 | 38.2 | 86 | 31.1 | 94 | no Cq | 98 | no Cq | 120 |
| 80 | - | - | no Cq | 118 | no Cq | 66 | no Cq | 82 | 32.9 | 122 | no Cq | 120 |
| 88 | - | - | 36.0 | 108 | no Cq | 86 | no Cq | 88 | 32.5 | 86 | - | - |
| 96 | - | - | no Cq | 57 | 37.1 | 56 | no Cq | 61 | 36.5 | 64 | no Cq | 54 |
| 99 | - | - | no Cq | 59 | 37.2 | 96 | no Cq | 79 | no Cq | 92 | no Cq | 51 |
| 106 | - | - | 30.0 | 53 | 29.3 | 61 | 30.1 | 58 | 30.5 | 80 | no Cq | 91 |
| 107 | - | - | no Cq | 51 | 37.1 | 73 | 31.3 | 64 | 34.0 | 99 | 37.1 | 103 |
| 119 | - | - | - | - | - | - | no Cq | 74 | no Cq | 115 | 32.8 | 93 |
| 120 | - | - | - | - | - | - | no Cq | 106 | no Cq | 122 | 36.4 | 70 |
| 121 | - | - | - | - | - | - | - | - | no Cq | 123 | 35.2 | 67 |
| 122 | - | - | - | - | - | - | - | - | no Cq | 114 | 36.5 | 95 |
| 123 | - | - | - | - | - | - | - | - | no Cq | 133 | 31.2 | 118 |
| 124 | - | - | - | - | - | - | - | - | no Cq | 134 | 35.6 | 93 |
| 125 | - | - | - | - | - | - | - | - | no Cq | 135 | 34.0 | 110 |
| 126 | - | - | - | - | - | - | - | - | no Cq | 123 | 31.8 | 61 |
| 128 | - | - | - | - | - | - | - | - | no Cq | 98 | 32.3 | 99 |
| 130 | - | - | - | - | - | - | - | - | no Cq | 132 | 34.4 | 70 |
| 132 | - | - | - | - | - | - | - | - | no Cq | 114 | 36.8 | 80 |
| **Thereof variable in cELISA** | | | | | | | | | | | | |
| 2 | - | - | no Cq | 81 | no Cq | 67 | no Cq | 67 | 35.0 | 23 | no Cq | 61 |
| 7 | - | - | 37.2 | 29 | 34.3 | 32 | 37.1 | 27 | no Cq | 58 | no Cq | 57 |
| 8 | - | - | no Cq | 69 | no Cq | 72 | no Cq | 65 | 35.8 | 12 | no Cq | 62 |
| 11 | - | - | no Cq | 90 | no Cq | 21 | no Cq | 75 | no Cq | 96 | 37.4 | 97 |
| 21 | - | - | no Cq | 26 | no Cq | 30 | no Cq | 40 | no Cq | 59 | no Cq | 7 |
| 25 | - | - | 37.0 | 46 | 34.2 | 50 | 36.3 | 57 | no Cq | 74 | no Cq | 73 |
| 34 | - | - | no Cq | 53 | 36.1 | 54 | 32.6 | 33 | 33.1 | 62 | no Cq | 77 |
| 46 | - | - | no Cq | 45 | no Cq | 45 | no Cq | 44 | 33.6 | 66 | 38.4 | 65 |
| 48 | - | - | no Cq | 43 | no Cq | 63 | no Cq | 65 | no Cq | 79 | - | - |
| 54 | - | - | no Cq | 20 | no Cq | 25 | no Cq | 27 | no Cq | 61 | no Cq | 49 |
| 70 | - | - | 38.1 | 50 | 35.9 | 44 | 34.0 | 40 | 33.8 | 55 | no Cq | 47 |
| 76 | - | - | 33.6 | 51 | 35.8 | 22 | 36.6 | 31 | 33.6 | 41 | no Cq | 52 |
| 79 | - | - | 37.9 | 36 | no Cq | 34 | no Cq | 42 | no Cq | 55 | no Cq | 61 |

| | | | | | | | | | | | | |
|---|---|---|---|---|---|---|---|---|---|---|---|---|
| 102 | - | - | no Cq | 43 | 38.2 | 60 | 30.6 | 66 | 32.3 | 27 | 34.5 | 4 |
| 114 | - | - | no Cq | 42 | no Cq | 60 | no Cq | 75 | no Cq | 107 | no Cq | 109 |
| 72 | 36.2 | 55 | no Cq | 33 | 35.3 | 32 | no Cq | 111 | no Cq | 139 | 37.1 | 93 |
| 127 | - | - | - | - | - | - | - | - | 32.2 | 28 | no Cq | 77 |
| 31 | - | - | no Cq | 92 | no Cq | 87 | no Cq | 92 | 35.7 | 37 | no Cq | 7 |
| 67 | - | - | no Cq | 55 | 36.8 | 40 | 34.0 | 10 | 36.9 | 39 | no Cq | 10 |
| 115 | - | - | 37.0 | 61 | 38.0 | 75 | 30.7 | 57 | no Cq | 59 | no Cq | 38 |
| **Thereof constantly positive in cELISA** | | | | | | | | | | | | |
| 10 | - | - | 34.4 | 25 | no Cq | 43 | 34.0 | 49 | no Cq | 43 | 36.3 | 45 |
| 43 | - | - | no Cq | 41 | 36.4 | 26 | 33.0 | 17 | 29.7 | 44 | - | - |
| 66 | - | - | no Cq | 25 | 37.3 | 23 | 38.4 | 19 | 33.8 | 32 | 38.8 | 26 |
| 71 | - | - | 38.0 | 10 | no Cq | 11 | no Cq | 8 | 35.2 | 11 | no Cq | 6 |
| 81 | - | - | no Cq | 17 | 34.7 | 19 | 38.0 | 13 | no Cq | 27 | 39.5 | 27 |
| 95 | - | - | no Cq | 14 | no Cq | 19 | 33.8 | 13 | 34.8 | 17 | 37.4 | 12 |
| 97 | - | - | 35.3 | 4 | 34.4 | 10 | 35.0 | 4 | 35.3 | 6 | no Cq | 4 |
| 69 | - | - | no Cq | 39 | - | - | - | - | - | - | - | - |
| 137 | - | - | - | - | - | - | - | - | - | - | no Cq | 16 |
| 138 | - | - | - | - | - | - | - | - | - | - | no Cq | 20 |
